# Computer-assisted surgery compared to fluoroscopy in curettage of atypical cartilaginous tumors / chondrosarcoma grade 1 in the long bones

**DOI:** 10.1371/journal.pone.0197033

**Published:** 2018-05-17

**Authors:** J. G. Gerbers, E. F. Dierselhuis, M. Stevens, J. J. W. Ploegmakers, S. K. Bulstra, P. C. Jutte

**Affiliations:** Department of Orthopaedics, University of Groningen, University Medical Center Groningen, Groningen, The Netherlands; Kanazawa University, JAPAN

## Abstract

**Introduction:**

Fluoroscopy is currently the standard imaging modality for curettage of atypical cartilaginous tumors/chondrosarcoma grade 1 (ACT/CS1). Computer-assisted surgery (CAS) is a possible alternative, offering higher resolution imaging and continuous three-dimensional feedback without ionizing radiation use. CAS hypothetically makes curettage more accurate, thereby decreasing residue or recurrence rate. This study aims to compare CAS and fluoroscopy in curettage of ACT/CS1.

**Patients and methods:**

A single center retrospective cohort study was performed. CAS and fluoroscopy were used in parallel. Included were patients who had curettage for ACT/CS1in the long bones, with a minimum follow-up of 24 months. Tumor volume was determined on pre-operative MRI scans. Outcome comprised local recurrence rates, residue rates, complications and procedure time.

**Results:**

Seventy-seven patients were included, 17 in the CAS cohort, 60 in the fluoroscopy cohort. Tumor volume was significantly larger in the CAS cohort (p = 0.04). There were no recurrences in either group. Residual tumor (2/17 vs. 7/60), complications did not differ significantly: fracture rate (3/17 vs. 6/60); nor did surgical time (1.26h vs. 1.34h).

**Discussion:**

CAS curettage showed good oncologic results. Outcome was comparable to fluoroscopy, while not using ionizing radiation. There was no significant difference in surgical time. Residue rates can likely be decreased with specific software functions and surgical tools.

## Introduction

Atypical cartilaginous tumor/chondrosarcoma grade one (ACT/CS1), ICD-O 9220/1, recently reclassified from chondrosarcoma grade one (CS-1), is one of the most frequently treated lesions in orthopedic oncology. [1] The most commonly affected sites are the diametaphysis of the proximal and distal femur, the proximal tibia and humerus. Incidence of chondrosarcoma as a whole was estimated in an analysis of the American Surveillance, Epidemiology and End Results (SEER) database as 1 in 200.000 per year. [2] A report of the European ESMO/EUROBONET registration describes the yearly incidence of chondrosarcoma as ~0.1/ 100.000. [3]

Because of ACT/CS-1’s potentially malignant nature, the surgical goal is a complete removal of the tumor to prevent local recurrences and its associated decrease in patient survival. [4,5] Up until around the 1980’s treatment of all chondrosarcoma consisted of resection with a wide margin. Better clinical and pathological knowledge and improved diagnostic techniques suggested this was not necessary for the less aggressive, low grade, lesions. The current standard surgical treatment therefore consists of (extended) intralesional curettage generally supported with fluoroscopy and the use of a local adjuvant such as phenol/ethanol, liquid nitrogen or argon beam coagulation.[6,7] Reconstruction is done with polymethylmethacrylate (PMMA), synthetic fillers, allografts or autografts. Depending on location and tumor characteristics, such as limited X-ray visibility due to cartilages’ lack of mineralization, it can be difficult to perform a complete curettage. The percentage of residual tumor after curettage is possibly significant, assuming that (early) local recurrence is often in fact local residue. [8]

Fluoroscopy, the current standard, offers two dimensional imaging and fluoro-video using X-band radiation. [9] Three-dimensional intra-operative imaging based on MRI may very well be an improvement in this aspect and there is no intra-operative radiation. With the advances of computer technology in the operating room, a new potential alternative has been developed. Computer-assisted surgery (CAS) is a relatively new modality, originally developed for neurosurgery in the early 1990’s. The main advantage of CAS over fluoroscopy is that it gives real-time, continuous, high resolution 3D feedback, all that and without the use of intra-operative ionizing radiation! It uses pre-operative computed tomography (CT) and/or magnetic resonance imaging (MRI) scans as visual datasets. Fusion of both modalities improves image clarity, especially for cartilage containing tumors. Tracked instruments are visible in the imaging environment. This means the surgeon is continuously aware of the 3D tumor and instruments location, with feedback on movement in three dimensions. In theory better orientation through CAS could make the surgery less demanding and improve outcome in recurrence and residue rates. Cited disadvantages for CAS use are lack of intra-operative assessment of the actual surgical result (i.e. the system shows a virtual result) and the system takes valuable surgical time to setup and configure. [10,11]

This study aims to compare fluoroscopy and CAS in terms of safety and efficacy in treatment of ACT/CS1 in the long bones.

## Patients and methods

### Design

A single center retrospective cohort study was performed using the prospectively kept local bone tumor database. All patients with the procedure code for curettages of bone tumors were analyzed. In accordance to regulations of the local Medical Ethical Review Board, all patients were informed about the fact that their data could be used for scientific research. If patients had objections to the use of their data these data were not included in the study.

### Patients

Inclusion criteria were: a curettage type procedure for histologically proven ACT/CS-1 in the long bones with the use of the adjuvants phenol and ethanol with a minimum follow-up of two years. Exclusion criteria were: the use of other means of treatment for the same lesion (e.g. radiofrequency ablation or cryotherapy), a non-complete follow-up and procedures that treated a recurrence. As this was a retrospective study patients were not randomized. This retrospective cohort study does not require an approval of the institutional review board (METc UMCG), following our research code. Patient approval is registered in the prospectively kept research database. Both techniques were used in parallel, with CAS use depending on system availability, planning and dataset quality.

Tumor volume approximation was done for each case on pre-operative MRI scans. The method used was as described by *Verdegaal et al*: calculation of the volume of an imaginary cylinder (π * r_max_^2^ * h_max_). For r_max_ the sum of maximum measured radii anterior-posterior and medial-lateral was divided by two to produce the maximum radius. We defined h_max_ as the largest measurement of proximal-distal size. [8]

### Outcome measures

The primary outcome measure was local residue or local recurrence. Local residue was defined as a suspect lesion (i.e. showing tumor like characteristics) reported on standard post-operative baseline imaging (MRI 3–6 months post-operative), with consensus between the radiologist and orthopedic surgeon. When there was no consensus an independent radiologist or orthopedic surgeon was consulted. Recurrence was defined as a positive pathological sample for ACT/CS-1 after a (radiologically) tumor-free period.

Secondary outcome measures were: complications like fractures, defined as a fracture at the surgical site regardless of adequate or inadequate trauma, other complications (eg. infection) and intra-operative and surgical time. Technique related time requirements were compared using the surgical time and patient-in-OR time. The surgical time was defined as the hours and minutes between first incision and wound closure as registered in the operative procedures registration database. Duration of the patient-in-OR time was defined as the period between the registered times of the patient entered the operating room and patient leaving the operating room.

### Patient work-up

Pre-operative workup included a CT scan (for the CAS navigation group), a gadolinium enhanced MRI with or without Short inversion-Time Inversion Recovery (STIR) fat suppression sequences. Core needle biopsies were performed to rule out grade 2 chondrosarcoma; they were done under CT-guidance and classified by one specialized musculoskeletal pathologist (AS) In case of earlier biopsy and referral, the material was revised by the pathologist. Pathology classification is standardized in the Netherlands by the Dutch Bone Tumor Committee, following the WHO classifications. [12] Surgical indication were clear diagnosis of ACT on MRI (e.g. septonodular Gadolinium enhancement, scalloping, wall-to-wall filling, perilesional oedema), growth of the tumor over time, and/or persistent pain on the tumor site The procedures were performed by two orthopedic surgeons (JP, PJ).

### CAS workflow

The curettages were done without pre-operative planning. Image fusion, generally CT with MRI, was done in the operating room while the patient was being prepared for surgery. The time consumption of tracker placement and software matching was measured using a stopwatch.

After bone exposure, the procedure differentiates from a standard (fluoroscopic) procedure [13]. During a navigated procedure a CAS patient tracker was rigidly attached to the affected bone using two 3 mm pins. Care was taken not to place the tracker in the expected path of the curette. Trackers were usually placed at percutaneously near joint lines, for example near the medial condyle of the femur, the anteromedial tibia plateau or the trochanteric complex. The pointer tool was then used for position checking, system calibration and remote control of the software. Image-based navigation was set-up by entering reference points both in the software and on the patient. The point based match was refined by surface matching where data points are entered with the pointer tool directly on the navigated bone. The system then presents an approximation of accuracy based on the difference between the entered points and the bone surface. The aim was an approximation of accuracy of lower than 1.0 mm. A Stryker Navigation System II with OrthoMap 3D software (Stryker Mahwah, NJ) was used in all cases. Surface matching on MRI is not supported on the used system. After the setup of the CAS the place for the bone window is determined using the pointer tool and the window is made in a regular fashion. The curettage technique, from a surgical point of view, is not different from a normal procedure; curettes are used to scrape out the lesion.

**Fig 1:**
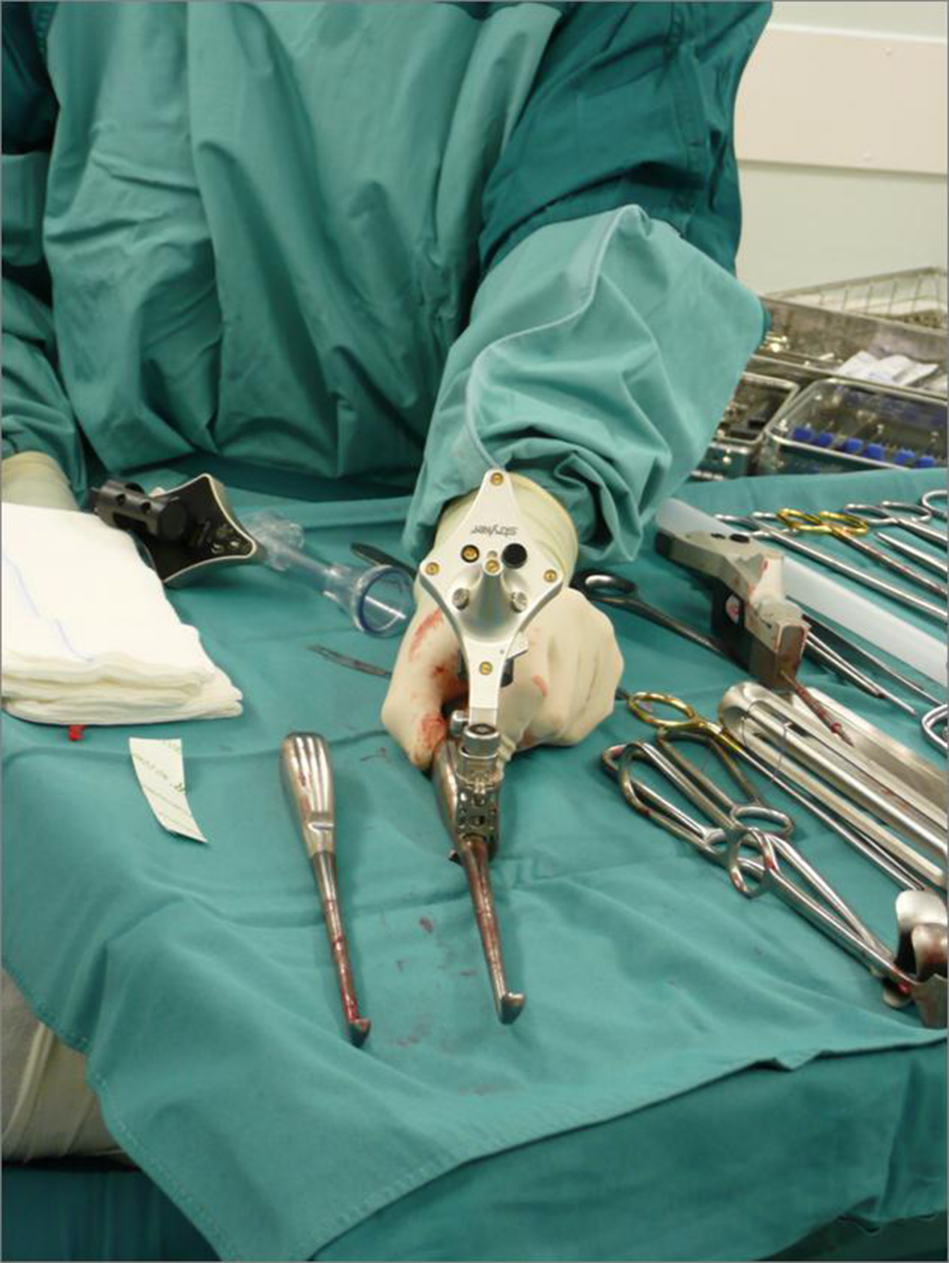
CAS tracker. Instrument tracker attached using a universal clamp to a large curette. Note the maximized three dimensional spacing of the infrared LED lights. The backside and battery compartment of another instrument tracker is visible in the background.

**Fig 2:**
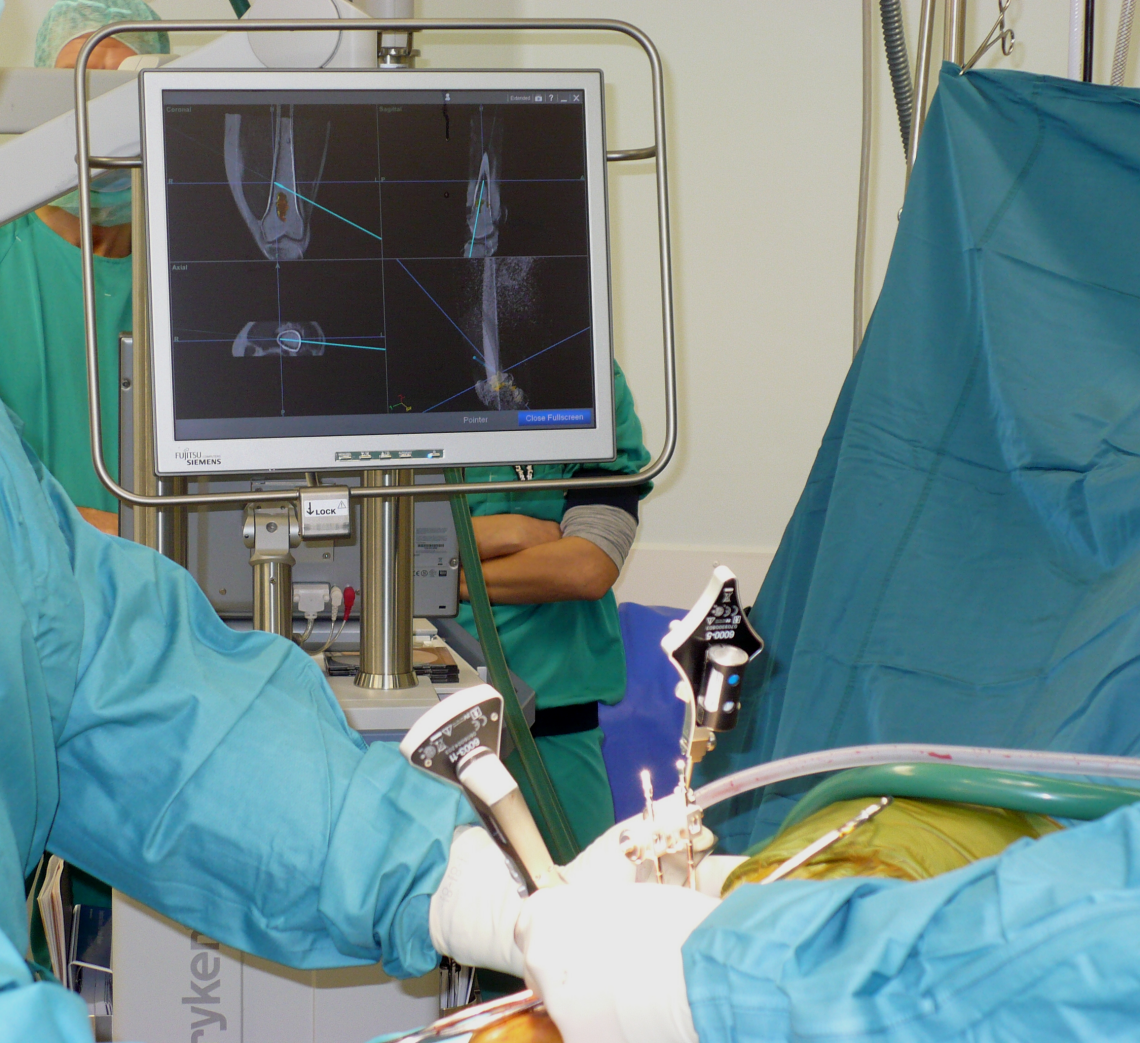
CAS procedure. Photograph during a typical CAS supported curettage procedure. Imagebased visualization mode is set to standard view. The system shows the relevant MRI slices, fused to the CT dataset, based on position of the tool. The ACT/CS1lesion is colored yellow by manual segmentation. The curette is visible as the blue line and the blue dotted line shows its vector in each direction. The lower right screen shows a volume render of the dataset, with the curette as the blue tool.

**Fig 3:**
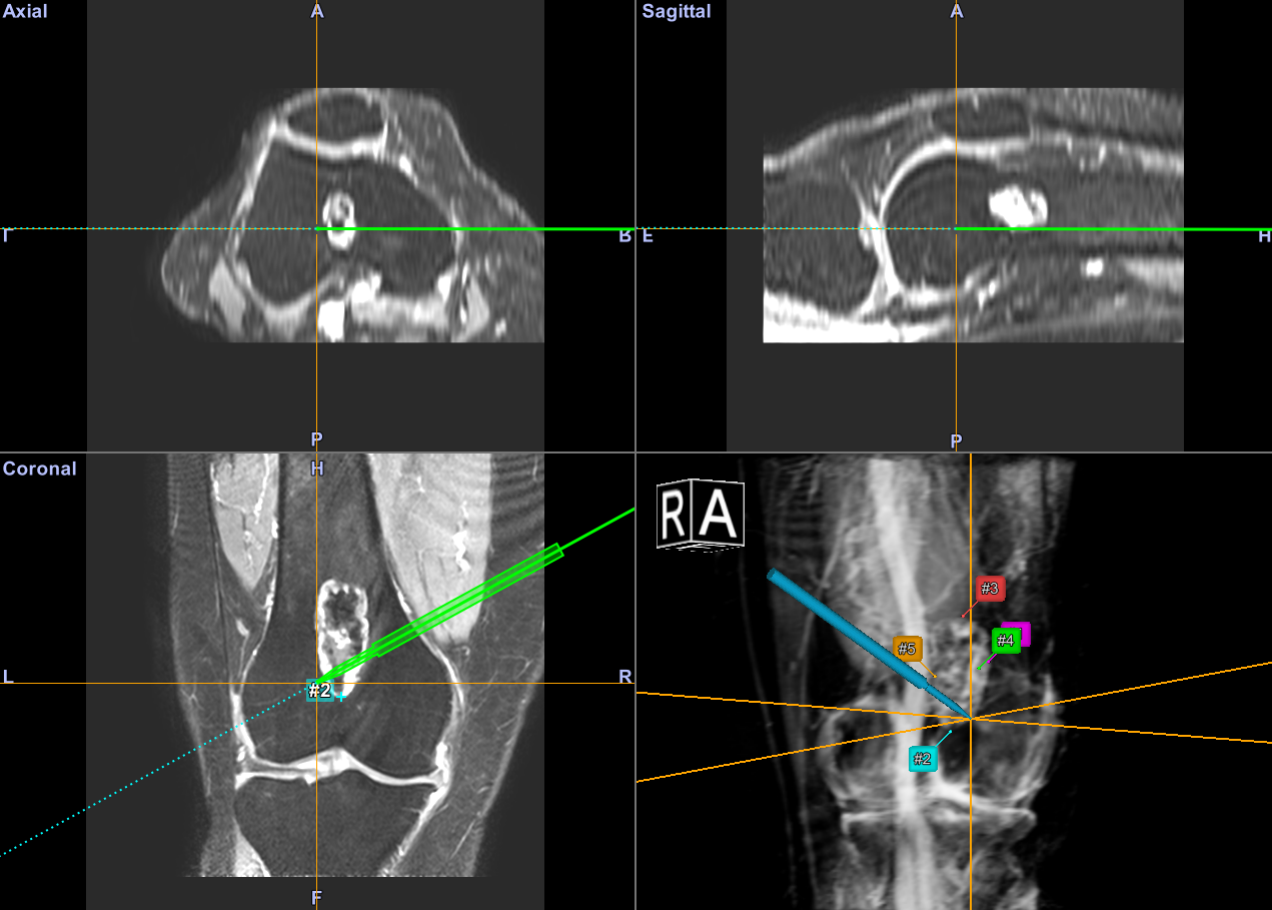
CAS workflow. Screenshot of the CAS interface during a final check of the curettage. An MRI dataset is used for navigation in this image. The pointer tool is inserted in the cavity and can extend beyond the tumor boundary. Annotation point marked by numbers are visible on the most extreme edges of the lesion as a further check.

The CAS system was used as a continuous-on imaging modality during the curettage process. A standard curette was attached to a tracker using a universal clamp and calibrated in the calibration device (Fig 1). During the procedure the situation on screen did not update as it was still based on pre-operative imaging data (Fig 2). A final check at the end of the procedure is performed by using the navigated curette to check the whole cavity: in all directions the pointer should access beyond the borders of the tumor (Fig 3). Screenshots were taken to register the extent of the curettage, comparable to a workflow with fluoroscopy. All CAS procedures were done without intra-operative fluoroscopy control.

### Fluoroscopy workflow

During a fluoroscopy supported curettage procedure imaging data was loaded onto digital displays in the OR for use during surgery. After dissection the lesion was localized with the fluoroscope to guide the place for the bone window. A window was made in a regular fashion in the cortex of the bone with an osteotome and hammer or electrical saw. The cavity was then curetted in a systematic clockwise way. Fluoroscopy was used for orientation during the procedure and to check if the curette reaches beyond all the visible edges of the tumor.

In both procedures the second part of the surgery is the same: when the curettage result was satisfying, the cavity was then partially filled with the first adjuvant: phenol. Small swabs were used to clean the edges of the whole cavity. The phenol was then washed out with 95% ethanol. The bone window was also cleaned using this protocol. The cavity was filled with PMMA or allograft bone chips. When indicated protective osteosynthesis material was applied to prevent a postoperative fracture (large window size, cortical resorption, diaphyseal localization). Plating was performed with a tibial LCP plate in diaphyseal lesions with at least two bicortical screws proximal and distal of the bone window. Weight bearing and return to activities depended on lesion size and location, usually it was 6 weeks partial weight bearing with crutches.

### Follow-up

After surgery, a standard X-ray was obtained as a routine post-operative check. Then a standard radiograph at the six weeks follow-up and at three to six months a baseline Gadolinium enhanced MRI. Further controls were yearly done with radiographs till year 3. Then a radiograph at year 5. When a residue was suspected or if the lesion appeared active, a more frequent MRI follow-up pattern was chosen. If the osteosynthesis material caused a too large interference for radiological analysis, even with MRI metal suppression protocols, CT scans were used.

### Statistical analyses

Descriptive statistics were used to describe the main characteristics of the patient groups. General patient data as age and sex were compared, depending on data type, using independent sample t-tests or Pearson chi-square tests. Specific categorical or dichotome variables, as recurrence and fracture rates, were compared with Fishers Exact test due to small sample sizes. Distribution of the different categories of reconstruction methods and the ASA classification were compared with the Freeman-Halton extension of Fisher's exact test, using the exact method.

Numerical surgical characteristics data were tested for normality using the Shapiro-Wilk test and were tested with a Student’s t-test. Non-continuous variables were compared with a Mann–Whitney U test. To assess potential causes for fractures binary logistic regression tests were performed with the dichotomous fractures as a dependent variable and calculated lesion size variable as continuous predictor and for the fracture rate as dependent variable and cement use as dichotomous predictor. All tests were done two-sided when applicable. A significance level of 0.05 was chosen. Analysis of the data was performed with IBM Statistical Package for the Social Sciences (SPSS) version 22.

## Results

### Patient characteristics

**Fig 4:**
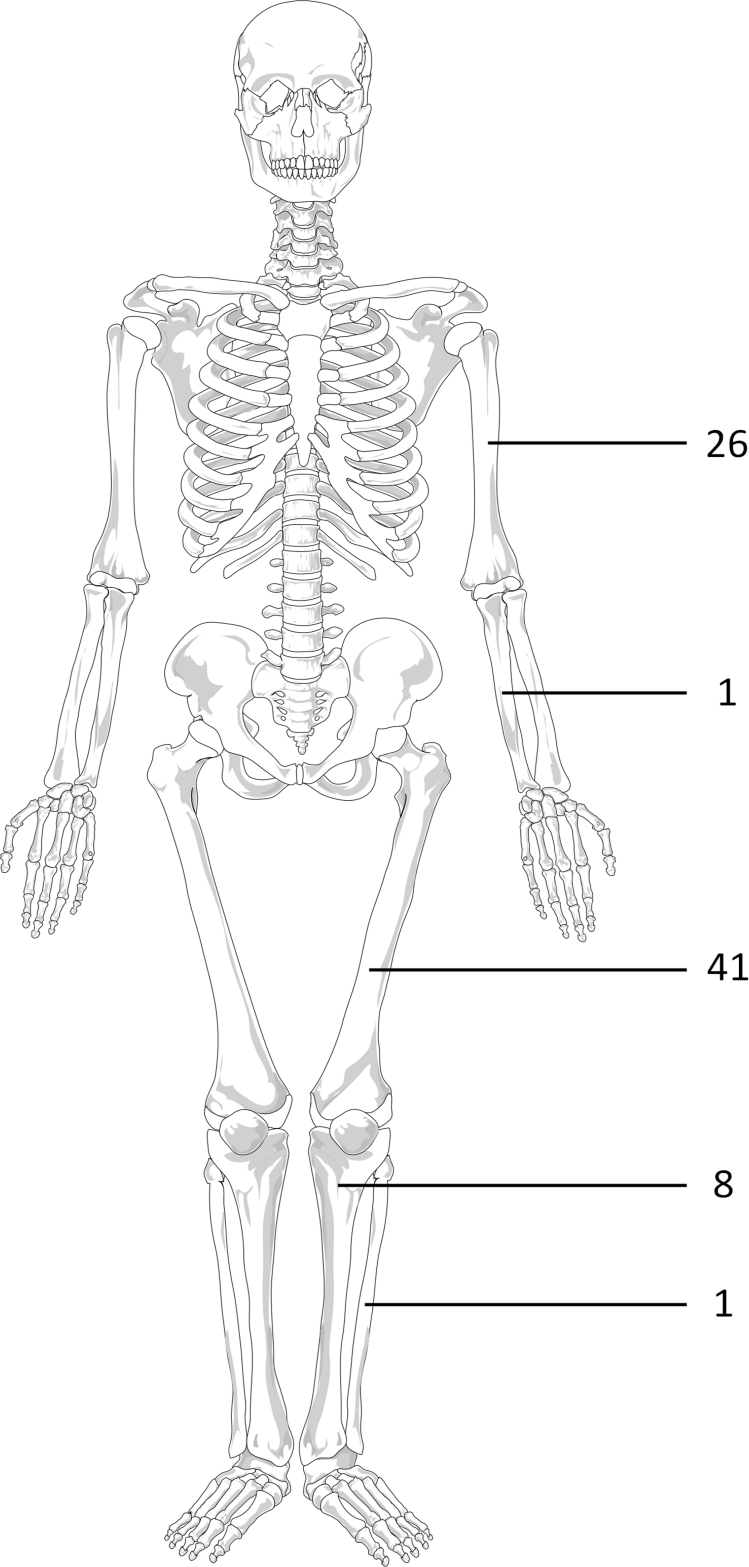
General distribution of location of the ACT/CS1 lesions.

Seventy-seven patients were included from 2006 to 2014; 17 patients in the CAS cohort and 60 patients in the fluoroscopy cohort. Mean age at surgery was 53 years (range 24–82 years). Females were slightly more affected than males with a ratio of 1.1:1. Median follow-up was 79 months (29–134 months, 50–134 for alive patients). Of the 77 patients included for analysis, 75 patients are currently alive; two patients have died of unrelated disease. Further demographic information is displayed in Table 1 and Fig 4.

**Table 1:** Demographic characteristics between the two cohorts.

	*CAS* *N = 17*	*Fluoroscopy* *N = 60*	*p value*
Patient age $\overset{-}{x}$ years)	56,1	53,8	0.50
Sex			>0.99
Male (n, %)	8 (47%)	29 (48%)	
Female (n, %)	9 (53%)	31 (52%)	
Follow-up $\overset{\sim }{x}$, months)	77	80	0.52

$\overset{-}{x}$ is cohort mean, $\overset{\sim }{x}$ is cohort median.

### Surgical characteristics

Analysis of the pre-operative data showed a significant difference between the lesion width (medial-lateral), with larger dimensions in the CAS cohort (p = 0.01). Median tumor volume was 18 cm^3^. Tumor volume was significantly larger (p = 0.04) in the CAS cohort; 23 cm^3^ (9 cm3–100 cm^3^) versus 16 cm^3^ (1 cm3–61 cm^3^) (see Table 2).

**Table 2:** Surgical characteristics between the two cohorts.

	*CAS*	*Fluoroscopy*	*p value*
ASA classification			0.92 *
ASA I (n, %)	6 (35%)	18 (30%)	
ASA II (n, %)	9 (53%)	35 (58%)	
ASA III (n, %)	2 (12%)	7 (12%)	
Lesion length ($\overset{\sim }{x}$ cm)	6.5	5.2	0.33 **
Lesion width ($\overset{-}{x}$ cm)	2.5	2.0	**0.01** *
Lesion depth ($\overset{-}{x}$ cm)	2.1	1.9	0.15 *
Calculated volume ($\overset{\sim }{x}$ cm^3^)	23	16	**0.04** **
Surgical time ($\overset{\sim }{x}$ h:mm)	1:26	1:34	0.29 **
OR time ($\overset{\sim }{x}$ h:mm)	2:15	2:27	0.24 **
Reconstruction			0.91 *
PMMA (n, %)	13 (76%)	45 (75%)	
Bonegraft (n, %)	4 (24%)	11 (18%)	
Synthetic graft (n, %)	0	3 (5%)	
None (n, %)	0	1 (2%)	

$\overset{-}{x}$ is cohort mean, $\overset{\sim }{x}$ is cohort median. Percentage displayed is percentage of cohort for that specific category.

*) Tested using Freeman-Halton extension of Fisher's exact test.

**) tested using Mann–Whitney U test, uniformly for length category.

Patient time in the operating room (patient-in-OR time) (2.15h versus 2.27h) and surgical time (1.26h versus 1.34h) was lower in the CAS cohort than in the fluoroscopy cohort, however both differences were not significant. CAS setup was measured in the last ten procedures from where the procedures deviates from the normal procedure (tracker placement) to the system fully set-up and running. This took on average 4 minutes and 25 seconds (range 2:03 min to 5:40 min).

### Clinical outcome

Nine patients, two in the CAS cohort (2/17, 12%) and seven in the fluoroscopy cohort (7/60, 12%) had a potential residue (p = NS). A more frequent follow-up strategy was initiated for these cases to check the potential residue for progression. Two of the potential residues have been biopsied, both in the CAS cohort. Both biopsies showed viable ACT/CS-1 and the residues were treated using radiofrequency ablation (RFA). There were no recurrences of the treated tumors in both cohorts (see Table 3).

**Table 3:** Clinical outcome.

	*CAS* *N = 17*	*Fluoroscopy* *N = 60*	*p value*
Recurrence (n, %)	0*	0**	-
(Potential) residue (n, %)	2 (12%)*	7 (12%)**	>0.99
Fractures (n, %)	3 (18%)	6 (10%)	0.41
Other complications (n, %)	0	5	0.58

Clinical outcome between the groups in events and percentage of that category. Complications were split in fractures and other complications. Other complications are split out in the text.

*) Two out of two have positive biopsies and have been treated using RFA.

**) No residues have been biopsied or treated.

There were nine fractures, related to the bone window, in the treated patients (9/77, 12%), all were within five months after the initial surgery and five were within one month. There were three fractures in the CAS group (3/17, 18%), all three in the diaphysis of respectively the femur (two) and humerus (one). There were six fractures in the fluoroscopy group (6/60, 10%). Five of these were in the diaphysis of the femur and one in the proximal metaphysic of the humerus. All were treated with osteosynthesis and are currently healed. The difference in fracture rate between the CAS and fluoroscopy cohort was not significant. There was no significant difference in the calculated tumor volume between the groups that had a fracture and those that did not (median of 19 cm^3^ versus 14 cm^3^). Prophylactic plating was done in 14 patients, although this could not prevent a fracture in two cases (12%).

There were eight fractures in the PMMA reconstruction group (8/58, 13.3%), none in the bonegraft group (0/15 patients, 0%), none in the synthetic bonegrafts group (0/3, 0%) and one in the no reconstruction group (1/1, 100%). Comparison of the tumor volume between PMMA and the non-PMMA groups showed no significant difference. The distribution of fractures over the groups was not significant (p = 0.1). In this dataset neither tumor volume, nor reconstruction method proved to be a predictive value for fractures in binary logistic regression. No complications were associated with either imaging modality.

## Discussion

Computer-assisted surgery has become an accepted treatment modality for difficult tumor resections.[11,14] While it offers potentially superior imaging feedback there have been no reports on use of CAS for the curettage of bone tumors, other than a few reported cases for bone tumors located in the spine. [15] The higher resolution imaging, three-dimensional feedback and no limitations in feedback time make CAS a potential alternative to fluoroscopy.

Clinical results of the CAS and fluoroscopy cohorts were comparable, with a significantly larger tumor volume in the CAS cohort. There were no tumor recurrences according to the definition; however nine residues were identified (13%). Although these outcome figures seem satisfying, they are difficult to compare to literature, were recurrence rates are reported between 3.5% and 13.3%, in studies with similar adjuvants. [16,17]. Important is that this depends on the interval and modality of follow-up imaging (i.e. MRI vs radiographs).

**Fig 5:**
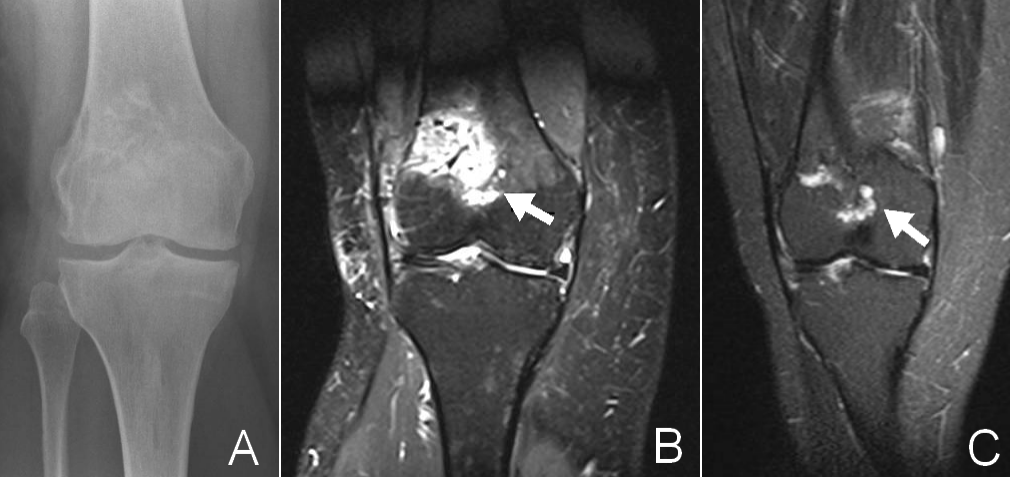
Residue. Series of images of a female patient after a CAS procedure for CHS1 in the right distal femur. A: the post-operative radiograph (Follow-up: 0 days). The defect is reconstructed with a bonechips (bonegraft). No residue visible. B: shows the baseline MRI scan of the same patient (follow-up 3 months). The depicted TIRM sequence shows a reactive response to the graft, with local edema. However below the reconstruction is another lobular, fluid rich structure: retrospectively suggestive forlocal residue but the radiologist describes it as most likely a postoperative reaction of the bone. Further radiographic follow-up shows no recurrence. C: TIRM sequence of the same patient (follow-up: 25 months) shows a nearly died down reaction to the graft but a comparable reaction in the suspected zone. Biopsy before RFA treatment confirmed CHS-1 tissue.

As *Verdegaal et al*. have demonstrated, some local recurrences might actually be residues. [8] ACT/CS-1 is a low grade tumor and generally grows slowly. Thus, suspect lesions on the 3 month baseline scans should be considered tumor residue. In fact all lesions detected within one year should probably be considered residues. Furthermore studies reporting recurrence rates using radiographs will likely miss smaller tumor residues. An example of a potentially missed residue can be seen in an image collage in Fig 5. While the post-operative radiographs of the knee show no apparent tumor residue the baseline MRI shows a suspect lesion. Taking this into account, recurrence will likely be over-reported and residue under-reported in studies using radiographs during follow-up. As residue is primarily a problem of intra-operative orientation, this is something the three-dimensional and high-resolution feedback aspect of CAS can possibly improve.

Potential residues were found in two out of 17 CAS patients and in seven out of 60 fluoroscopy patients. Both potential residue cases in the CAS cohorts and one out of the fluoroscopy cohort showed tumor tissue at biopsy. Actual residue rate for the fluoroscopy cohort may be lower than six as GD-MRI has positive prediction rate of 45% for actual residue on suspect follow-up scans. [8] Analysis of the CAS residue cases did not show a clear cause. Our hypothesis is that the feedback mode is currently not yet good enough for CAS to be better than fluoroscopy, especially the lack of progress tracking (i.e. there is no change in the image dataset on screen during the procedure). It also shows that it’s not an infallible guide. While no navigational inaccuracies were experienced, regular accuracy checks, on a known landmark, are advised during the procedure to prevent drift.

Fracture rates in this study (12%) are high compared to literature. Recent studies, with the same adjuvants, show a fracture rate ranging from 5.0 to 6.6 percent. A possible cause for this can be difference in preventive osteosynthesis strategies. [7,17–21] 58 of the 77 patients treated in this study had a reconstruction with PMMA. While it is suspected that the exothermic PMMA hardening process can have a beneficial effect as an adjuvant, it may have an negative effect on the host bone. Literature shows no significant difference in recurrence and fracture rates between using PMMA or other reconstruction methods. [17,22] As there were no recurrences, we cannot draw conclusions on PMMA and recurrence rate. Fracture rate in PMMA seems higher with 8 fractures out of 58 patient treated (14%) compared with a 6% fracture rate in the other reconstruction methods, although this did not reach significance. As the fracture rate was considered too high, a more aggressive plating strategy (longer plates and more cortices for smaller lesions) was adopted for procedures in the diaphysis of the femur. There was also a possible bias in fracture risk as a concurrent radiofrequency ablation (RFA) trial meant the exclusion of patients with mainly smaller ACT/CS-1 lesions in the femoral metaphysis. [23]

The often cited downside of CAS use, the long set-up time, was not experienced. [10] Set-up time, measured in the later cases, was on average just 4 minutes and 25 seconds. This was with an experienced team with over 50 procedures into the learning curve. The median surgical and patient in OR time was less than for fluoroscopy. While not significantly better, it shows that the set-up time is recouped during the procedure.

Use of CAS has been described by both surgeons as useful, due to the continuous three dimensional feedback, compared with the intermitted two dimensional feedback in fluoroscopy. It was helpful in checking for complete removal in difficult zones for example directly around the cortical window. Application of CAS in the humerus was considered more difficult due to issues with tracker placement in the working field. Smaller trackers could solve this issue. There were no direct complications nor any morbidity related to use of the CAS system. Possible complications as pin tract fractures or pin tract infections did not occur.

Inter-observer variability is a high and ongoing issue in the grading of cartilaginous bone tumors. The SLICED study group reported that grading reliability, even by experienced pathologists and radiologist, is low and that this may partially explain difference in outcomes between centers [24]. The difference is even highest between discerning enchondroma and low grade chondrosarcoma (kappa 0.54) [25]. In this study one specialized pathologist with extensive expertise in sarcoma and two specialized radiologist either reviewed the samples or supervised a resident for nearly all the cases. All external work was reviewed. Guidelines on classification for ACT/CS1 were rigidly followed.

This study has some limitations. It was set-up as a retrospective cohort study, the study population was not equally divided and the techniques were not actively randomized. Both the techniques were however used in parallel, with CAS use only depending on system availability, planning and dataset quality. The only adjuvant treatment in this study was phenol/ethanol, results may be different using argon beam coagulation or cryotherapy as adjuvant. CAS curettage was tested only for ACT/CS1, the effect on lesions with higher recurrence rates as giant cell tumor may be different. As far as we know, this is the first study on the usage of CAS for curettage of ACT/CS-1 in the long bones. This study can be seen as a pilot study on CAS efficacy.

Some improvements to workflow and instruments will probably have a positive effect on outcome measurements. Currently it’s not possible to see the extent of the already treated area. A ‘paintbrush’ mode, where the position of the tip is painted into the three-dimensional view would provide feedback on surgical progress. This together with the addition of a planning mode to the software could provide an intra-operative residue check (i.e. coloring in the planned 3d structure). Furthermore it is likely that a more accurate curettage with less healthy bone removed can decrease fracture rates. Also, there should be support for non-straight tools like bended, hockey stick, shaped curettes and pointers, for easier access to tumor tissue in corners of the lesion.

## Conclusion

CAS curettage with phenol/ethanol adjuvants has shown good oncological results at medium length follow-up, at least comparable to the fluoroscopy cohort and literature. CAS curettage in this study was safe and effective. There were no recurrences, and no difference in the occurrence of residues between the cohorts, this despite significantly larger lesions in the CAS cohort. Fracture rates in both groups were higher than expected. In this study this was not linked to CAS technique, PMMA use or size of the lesions.

Especially a suspected high residue rate after curettage supports the development of better intra-operative orientation. Clinical outcome of the present study supports CAS use as an alternative to fluoroscopy. With CAS no ionizing radiation was used during these surgeries and there was no increase in surgical time. Residue rates can likely be improved with specific, curettage targeted, software modules and tools.

-

## Supporting information

S1 Dataset Anonymized study data. (SAV)
